# A Weld Position Recognition Method Based on Directional and Structured Light Information Fusion in Multi-Layer/Multi-Pass Welding

**DOI:** 10.3390/s18010129

**Published:** 2018-01-05

**Authors:** Jinle Zeng, Baohua Chang, Dong Du, Li Wang, Shuhe Chang, Guodong Peng, Wenzhu Wang

**Affiliations:** 1 Key Laboratory for Advanced Materials Processing Technology, Ministry of Education, Department of Mechanical Engineering, Tsinghua University, Beijing 100084, China; zengjinle@casicloud.cn (J.Z.); bhchang@tsinghua.edu.cn (B.C.); wanglidme@tsinghua.edu.cn (L.W.); changsh15@mails.tsinghua.edu.cn (S.C.); pgd16@mails.tsinghua.edu.cn (G.P.); wwz13@mails.tsinghua.edu.cn (W.W.); 2 Beijing Aerospace Smart Manufacturing Technology Development Co., Ltd., Beijing 100039, China

**Keywords:** multi-layer/multi-pass welding, seam tracking, visual detection, information fusion

## Abstract

Multi-layer/multi-pass welding (MLMPW) technology is widely used in the energy industry to join thick components. During automatic welding using robots or other actuators, it is very important to recognize the actual weld pass position using visual methods, which can then be used not only to perform reasonable path planning for actuators, but also to correct any deviations between the welding torch and the weld pass position in real time. However, due to the small geometrical differences between adjacent weld passes, existing weld position recognition technologies such as structured light methods are not suitable for weld position detection in MLMPW. This paper proposes a novel method for weld position detection, which fuses various kinds of information in MLMPW. First, a synchronous acquisition method is developed to obtain various kinds of visual information when directional light and structured light sources are on, respectively. Then, interferences are eliminated by fusing adjacent images. Finally, the information from directional and structured light images is fused to obtain the 3D positions of the weld passes. Experiment results show that each process can be done in 30 ms and the deviation is less than 0.6 mm. The proposed method can be used for automatic path planning and seam tracking in the robotic MLMPW process as well as electron beam freeform fabrication process.

## 1. Introduction

**Figure 1:**
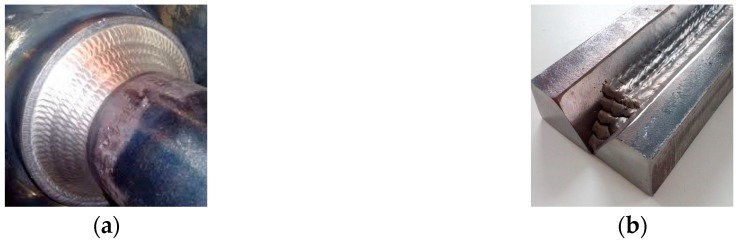
Examples of the multi-layer/multi-pass welding components with large wall thickness. (**a**) A welded pipe component; (**b**) A welded plate component.

Welding is widely used in the energy industry to join the thick components, such as hydraulic turbine blades, power plant pipelines, and steam generators and low pressure rotors in nuclear engineering. These components are usually large and thick, and must be welded by multi-layer/multi-pass welding (MLMPW) technology (Figure 1) [1]. Nowadays, lots of automatic welding equipment such as welding robots are used to perform accurate welding processes. For better welding quality, it is essential to perform a reasonable path planning for each weld pass at each layer before welding, so that the motion actuators can move along the welding path accurately during the welding process. However, there are usually some inevitable machining errors, assembly errors, and thermal deformations in the workpieces during welding; moreover, the geometry of the weld beads formed may not be as regular as expected. These factors may cause the deviations between the predetermined planning path and the actual weld pass position. Therefore, it is very important to recognize the actual weld path automatically during welding, based on which the motion path of the actuators can be adjusted accordingly to correct the path deviations in real time [2,3,4,5,6,7].

**Figure 2:**
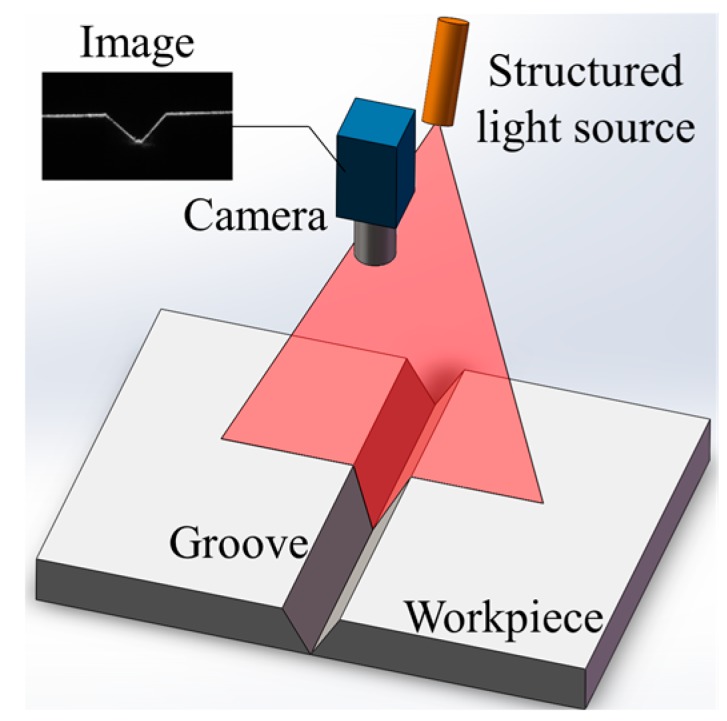
The structured light method applied to V-groove detection. The distortion of the laser stripe mirrors the geometrical information of the groove.

Visual detection methods have the advantages of abundant information, no contact with the workpiece, good sensitivity, and high precision, and are considered some of the most promising weld position recognition technologies. Nowadays, structured light visual detection methods are widely used in industry and many practical sensors having been developed by several companies, such as Meta Vision, Servo Robot, and Scansonic [8,9,10,11,12]. In this method, the distortion of a projected laser stripe mirrors the geometrical differences between the base metal and the groove/bead (Figure 2).

Based on the triangulation technique, the welding path can be detected accurately when there are significant geometrical differences between the base metal and the groove/bead. Relevant image processing methods have been proposed by many researchers to detect the weld positions [13], as shown in Table 1.

**Figure 3:**
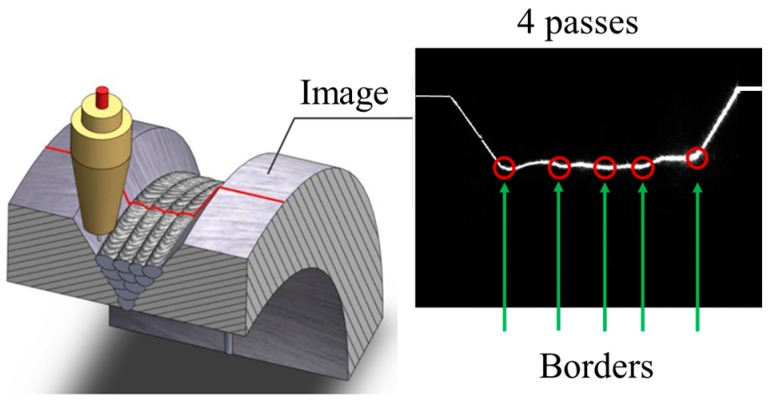
The structured light method applied to MLMPW detection. The distortion of the laser stripe is small between adjacent passes.

However, the structured light detection method may fail when detecting the MLMPW positions [17,28]. Although many algorithms have been proposed to obtain the MLMPW positions based on structured light images [29,30,31], the structured light method is not applicable to MLMPW detection due to the small geometrical differences between adjacent passes, as shown in Figure 3.

**Figure 4:**
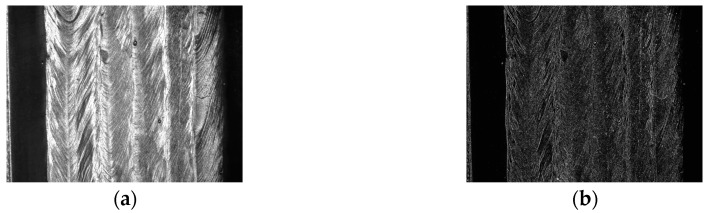

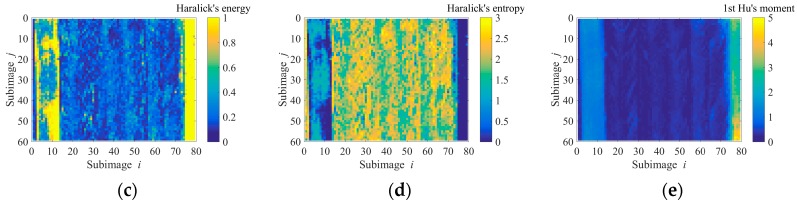
The distributions of the grayscale gradient and texture features in MLMPW image. (**a**) An example of the MLMPW image; (**b**) The grayscale gradient distribution using Sobel operator; (**c**) The Haralick’s energy distribution; (**d**) The Haralick’s entropy distribution; (**e**) The first Hu’s moment distribution.

Besides the structured light method, there are many other weld position detection methods based on various visual features, such as grayscale gradient and texture features. For example, Du et al. proposed that the Haralick’s texture features such as “energy” and “entropy” are greatly different between the base metal and the beads, which can be used for weld position detection [32]. Zeng et al. studied the Hu’s invariant moments and found that compared with the Haralick’s texture features, the first invariant moment has higher separation degree in weld position detection fields [33]. Zou et al. fused the grayscale gradient information and the structured light image using conference-weighted method, which achieved an accurate detection result when the structured light distortion is small [34]. Krämer applied the texture features in T-joint position recognition using machine learning methods, and obtained a satisfactory detection result [35]. The studies mentioned above may give accurate results when there is no or only one weld pass, but no relevant research has been reported in multi-pass detection fields. In addition, most of these methods can only obtain 2D path information using the grayscale image of the workpiece, which indicates that they may not be able to distinguish the weld passes in different layers. Moreover, the distributions of the grayscale gradient and texture features are usually so non-uniform and complex that it is hard to distinguish the borders of the adjacent weld passes, as shown in Figure 4. Overall, there is no suitable detection method in the MLMPW recognition fields to date.

In this paper, a novel detection method for MLMPW recognition is proposed, which fuses the information from both the directional light image and the structured light image. The synchronous acquisition method is studied to obtain different images for different lighting conditions, and an information fusion method is proposed to recognize the 3D weld pass position. In Section 2, the experimental platform used in the study is introduced. Then, the synchronous acquisition method is proposed using the trigger signals in Section 3. Before weld position recognition, the interferences from arc light and spatters in the captured images are eliminated by fusing adjacent images, as detailed in Section 4. In Section 5, the processing methods are presented to obtain an approximate weld position recognized when the directional light is used. In Section 6, the structured light images are processed to obtain candidate positions of the bead borders. The information fusion method is proposed in Section 7 to obtain the 3D positions of the weld passes, which combines different visual features extracted from directional and structured light images. Experiments carried out to examine the applicability of the proposed methods are introduced in Section 8. Finally, conclusions are summarized in Section 9.

## 2. Configuration of the Experiment Platform

**Figure 5:**
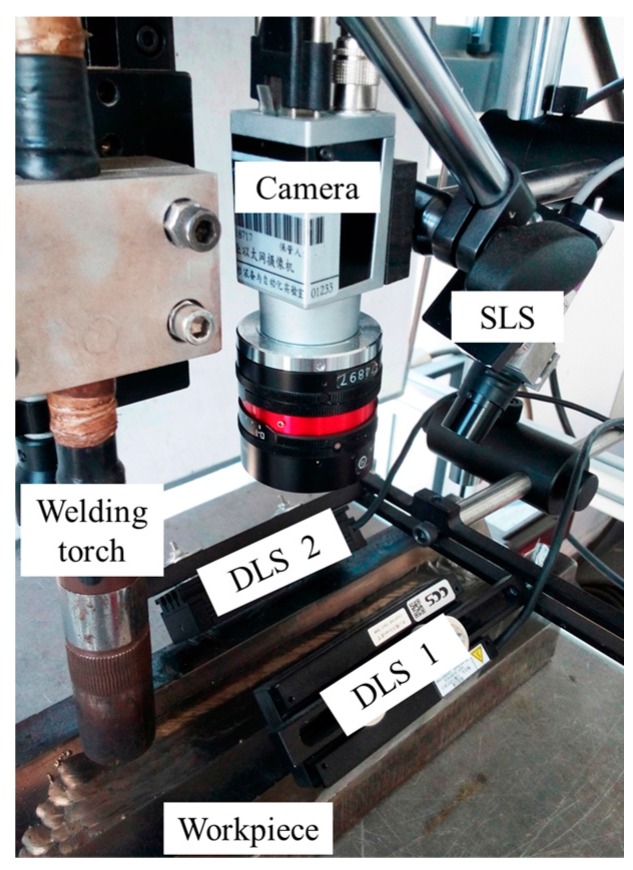
The experiment platform used in the study.

The experimental platform is shown in Figure 5. It comprises a MIG/MAG welding torch, a camera, two directional light sources (DLS), a structured light source (SLS), a S7-200 PLC (Siemens, Berlin and Munich, Germany), and an industrial computer. The camera is an acA1600-60gm (Basler, Ahrensburg, Germany) with 60 fps maximum frame rate at 1200 × 1600 maximum size. Both of the DLSs are LDL2-74X30RD (CCS, Kyoto, Japan), symmetrically mounted on both sides above the workpiece.

The power and wavelength of the SLS are 50 mW and 635 nm, respectively. The central wavelength of the DLSs is 630 nm. An optical filter with 635 nm central wavelength and 10 nm FWHM (full width at half maximum) is mounted on the lens of the camera to filter out the arc light. The workpiece is placed on a translational stage to move during the welding process. The distance between the center of the camera FOV and the welding torch is about 45 mm. The distances between the workpiece and the DLSs are about 30 mm. We denote the two DLSs and the SLS by *L*_1_, *L*_2_ and *L*_3_, respectively. The industrial computer is used for image processing. The CPU frequency and RAM size of the industrial computer are 2.3 GHz and 4 GB, respectively.

## 3. The Synchronous Acquisition Method to Capture Various Kinds of Images

**Figure 6:**
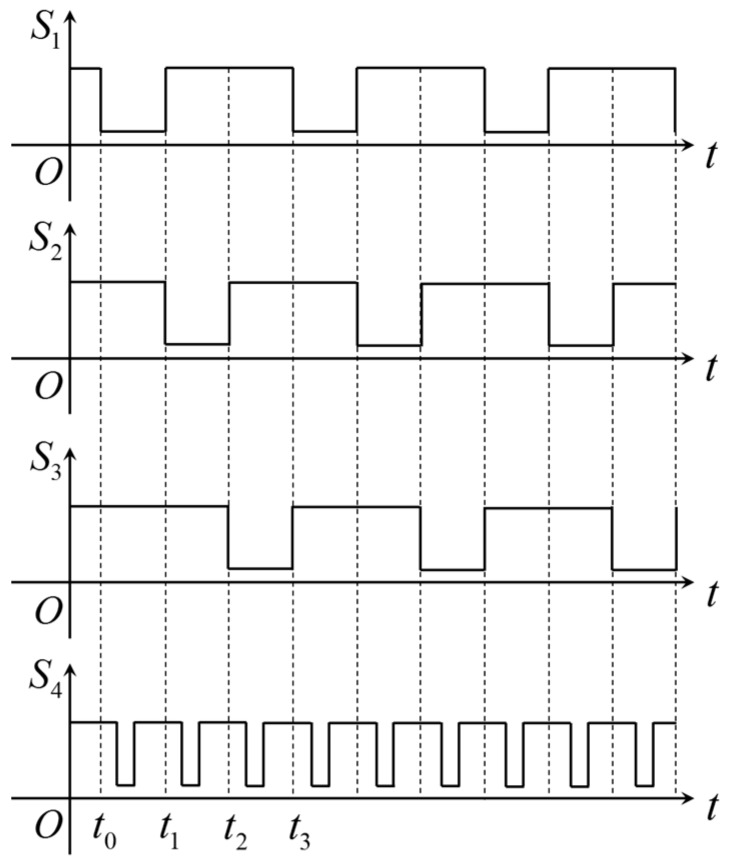
The timing diagrams of the trigger signals generated by S7-200 PLC (Siemens, Berlin and Munich, Germany).

The PLC in the experimental platform is used to generate four trigger signals *S*_1_–*S*_4_ to synchronize *L*_1_, *L*_2_, *L*_3_ and the camera shutter. The timing diagrams of the trigger signals are shown in Figure 6, in which the light sources and camera are enabled to function only in the low-level periods of their trigger signals.

**Figure 7:**
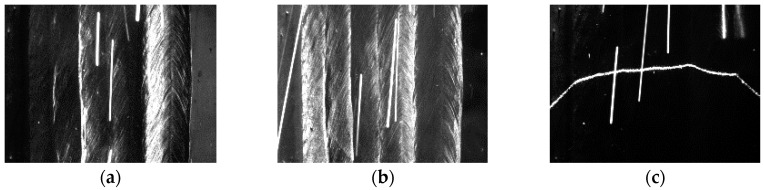
The captured images in different lighting conditions. (**a**) The left-side DLS enabled; (**b**) The right-side DLS enabled; (**c**) The SLS enabled.

According to Figure 6, the light sources *L*_1_, *L*_2_ and *L*_3_ are switched on one by one, and the camera is synchronized to capture images for each light source. Figure 7 shows an example of the captured images in adjacent frames with different lighting conditions. The frame rate of the camera is set to 30 fps. In this paper, the borders between the weld passes will be recognized using these three kinds of images.

## 4. Elimination of the Interferences from Arc Light and Spatters by Fusing Adjacent Images

The arc light and spatters both increase the grayscales of certain regions, and therefore must be eliminated for accurate weld position detection. The intensity distributions of the arc light and spatters in the image are time-varying, so they can be removed by fusing adjacent images at pixel level. Denote the grayscales of the image captured at time *t_n_* by *I*_0,*n*_(*x*,*y*) when one of the light sources enabled. The denoised image *I_n_*(*x*,*y*) can be calculated by:

(1) $$I_{n}\;\left(x,y\right)=\min\;\left\{I_{0,n},\;\left(x,y\right),\;I_{0,n-1},\left(x,\;y\right),\cdots ,\;I_{0,n-\left(k-1\right)}\left(x,y\right)\right\},$$

where *k* is the selected image number, and *I*_0,*n*_ (*x*,*y*), *I*_0,*n*−1_ (*x*,*y*), …, *I*_0,*n*−(*k*−1)_ (*x*,*y*) are the captured images when the same light source is enabled in its adjacent trigger units.

When *L*_1_ or *L*_2_ is enabled, the grayscales of the denoised image calculated by Equation (1) may be extremely low, even if *k* = 2. This is because the grayscale distribution of the images is quite non-uniform: the regions *R_H_* with high grayscales may be surrounded by the regions *R_L_* with low grayscales, and when Equation (1) is used, the positions of *R_H_* and *R_L_* in adjacent images may overlap each other due to the movement of the workpiece during welding. Therefore, the grayscale values of the denoised images may seriously decrease if only (1) is used. In this paper, first set *k* = 2 and use (1) to obtain the denoised image *I*_1,*n*_(*x*,*y*). Then in order to keep the overall grayscale levels of the images, the final denoised image *I_n_*(*x*,*y*) is obtained by choosing the maximum grayscale values of the corresponding pixels in adjacent denoised images *I*_1,*n*_(*x*,*y*), i.e.,:

(2) $$I_{n}\;\left(x,y\right)=\max\;\left\{\min\;\left\{\;I_{0,n}\;\left(x,y\right),\;I_{0,n-1}\;\left(x,\;y\right)\right\},\;\min\;\left\{\;I_{0,n-1}\;\left(x,y\right),\;I_{0,n-2}\;\left(x,y\right)\right\}\right\},$$

**Figure 8:**
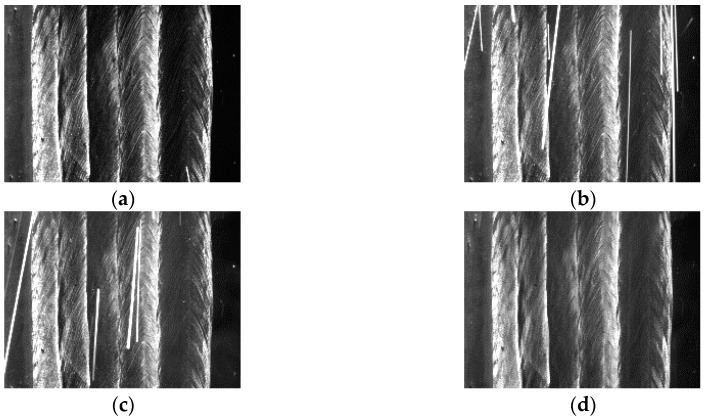
The denoised results when *L*_2_ is enabled. (**a**) Image captured at time *t_n_*_−2_; (**b**) Image captured at time *t_n_*_−1_; (**c**) Image captured at time *t_n_*; (**d**) The final denoised image.

**Figure 9:**
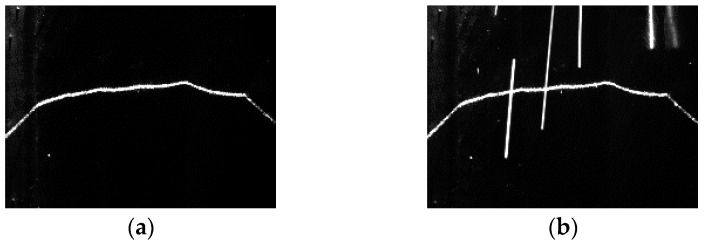

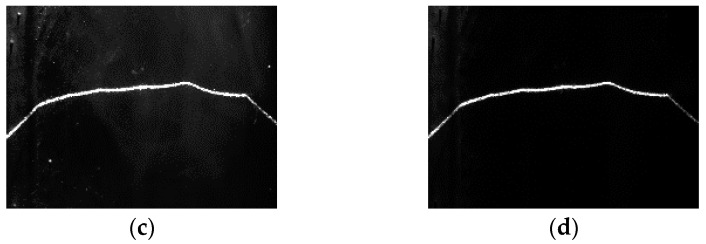
The denoised results when *L*_3_ is enabled. (**a**) Image captured at time *t_n_*_−2_; (**b**) Image captured at time *t_n_*_−1_; (**c**) Image captured at time *t_n_*; (**d**) The final denoised image.

The denoised results of Equation (2) are shown in Figure 8. The interferences from the arc light and spatters have been completely eliminated and the overall grayscale levels have been kept well. When *L*_3_ is enabled, set *k* = 3 and use Equation (1) to eliminate the arc light and spatters. The denoised results are shown in Figure 9. The arc light and spatters are both completely eliminated too.

## 5. Processing Method When the Directional Light Source Is Enabled

**Figure 10:**
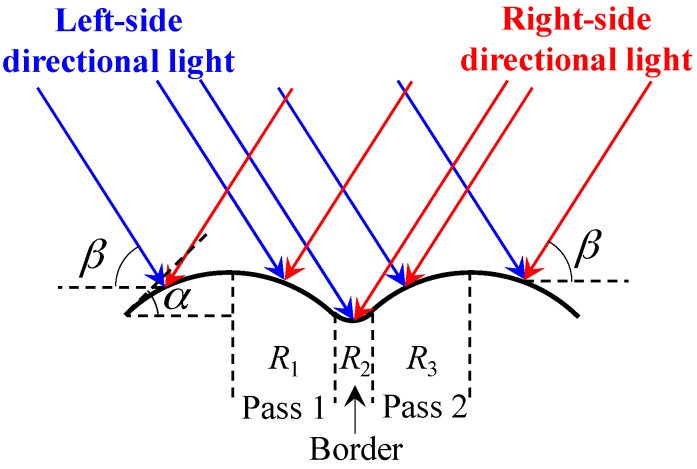
The schematic diagram showing the directional lights irradiated on the surface of a workpiece, where the light in blue is from the left-side directional light source and the light in red is from the right-side directional light source.

Figure 10 shows the scene when the directional light is projected onto the weld bead surface. There are always tiny protrusions on the bead surface due to the surface tension in the welding process. Based on the principles of geometrical optics, the irradiance ratio between the region *R*_1_ and *R*_3_ in Figure 10 is about sin(*β* − *α*)/sin(*β* + *α*) < 1 when the left-side directional light is on, where *β* is the projection angle of the directional light and *α* is the angle of the welding toe. It indicates that the grayscale of region *R*_1_ is slightly smaller than the grayscale of region *R*_3_. Similarly, the grayscale of region *R*_1_ is slightly larger than the grayscale of region *R*_3_ when the right-side directional light is on. The fusion zone between adjacent passes, i.e., the region *R*_2_ in Figure 10 is relatively smooth, so that the grayscales are almost the same when the left-side or right-side directional light is on.

In this paper, the image captured when *L*_1_ is on is subtract from the image capture when *L*_2_ is on, and the absolute differential image is denoted by *K_n_*(*x*,*y*). According to the analysis mentioned above, the grayscale of region *R*_1_ and *R*_3_ in *K_n_*(*x*,*y*) would be larger than the grayscale of region *R*_2_. Therefore, there should exist a “high grayscale to low grayscale to high grayscale” (HLH) region near the borders between adjacent passes in *K_n_*(*x*,*y*). In reality, the geometrical morphology of an actual bead surface could be highly non-uniform, and the grayscale of region *R*_1_ would be larger than *R*_3_ when the left-side directional light is on. However, as long as there are tiny geometrical differences between adjacent passes and the fusion zone is smooth, the HLH region would exist in the absolute differential image *K_n_*(*x*,*y*).

**Figure 11:**
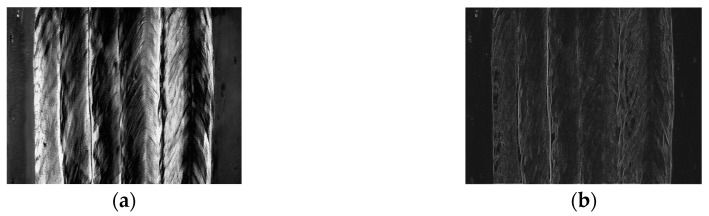
The absolute differential image and its gradient distribution. (**a**) The absolute differential image; (**b**) The gradient distribution of the absolute differential image obtained by using Sobel operator.

Figure 11a shows the absolute differential image *K_n_*(*x*,*y*), and it can be seen that the HLH regions do exist near the borders between adjacent passes. In addition, since the angle between the groove sidewall and the directional light is quite different from the angle between the bead and the directional light, there would be some regions with large grayscale gradients near the borders between the beads and the groove sidewalls. The absolute differential image (Figure 11a) shows these regions too.

Figure 11b shows the gradient distribution of absolute differential image obtained by using Sobel operator. The gradients near the borders are large, but may not be larger than other large gradient values caused by the bead texture. Therefore, traditional thresholding methods cannot be used directly to detect these kinds of weak borders.

**Figure 12:**
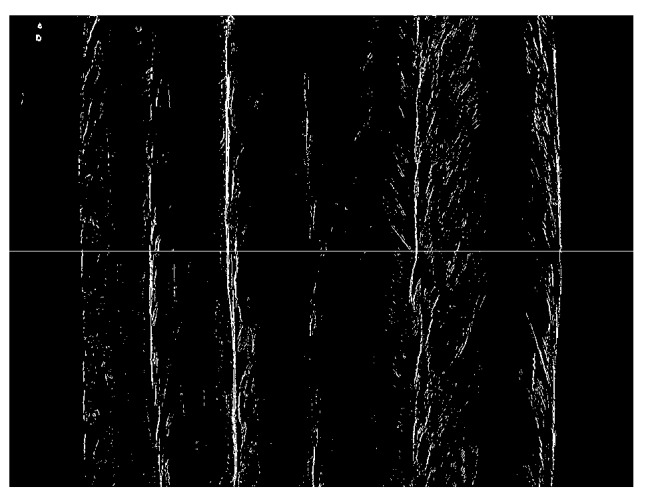
The resulted image after choosing 50 points with largest gradient values in each row.

In this paper, it is proposed to scan each row of the gradient image at first to find *N* points with largest gradient values in each row. *N* must be much larger than the number of passes, so that most of the points on the weak borders are retained in this step. In this paper, *N* of 50 is used, which is ten times the number of passes. The detection result is shown in Figure 12. Then, the number of pixels is counted in each column of Figure 12, and the low-pass filtering is applied to the counting results.

**Figure 13:**
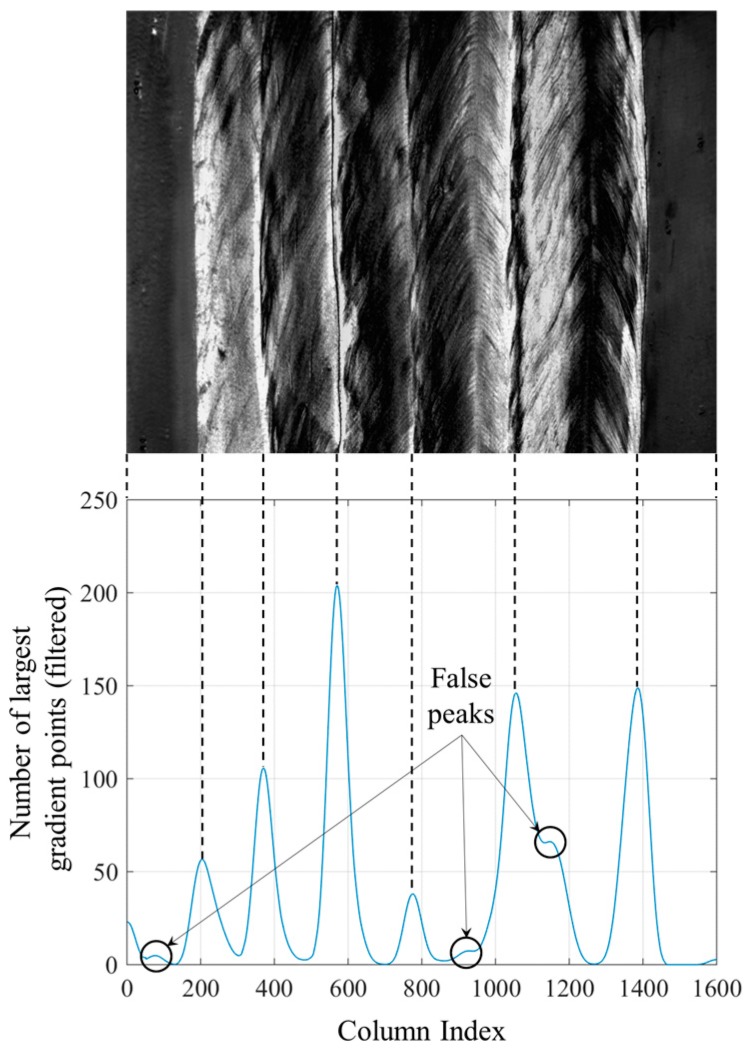
Number of largest gradient points after filtering. The valid peaks in the curve correspond to the borders between weld passes.

Figure 13 shows the filtered results. The low-pass filter is a 101-point FIR filter designed by window design method using Hamming window function. The cut-off frequency of the FIR filter is 0.01. Except some false peaks, each valid peak in Figure 13 corresponds to one of the borders. Although the pixels on the same border are not located in the same column as shown in Figure 12, the filtered counting result of each column still mirrors the positions of the borders.

False peaks in Figure 13 must be eliminated using proper methods. In this paper, the peaks were removed at first, of which the peak-to-peak values are less than 10% of the maximum value; then a non-maximum suppression algorithm is applied to eliminate the peaks close to each other. The distance threshold chosen in non-maximum suppression algorithm should be less than the minimum width of the passes, otherwise the weld pass with a width less than the distance threshold would not be detected. In this paper, the minimum width of the passes is more than 50 pixels, so the distance threshold is set to be 50 pixels. After these two steps, only six valid peaks are left in Figure 13, which all correspond to the positions near the actual borders.

Using the images when the directional light source is enabled, the approximate positions of the borders have been obtained. But, there are still some deviations between the detection results and the actual positions of borders. In addition, since only the 2D information of the MLMPW paths can be obtained, the passes in different layers cannot be distinguished. In contrast, the structured light images can reveal the 3D information of the beads, but the accurate positions of the weld passes cannot be detect. Therefore, these two kinds of information are fused to obtain the accurate 3D weld position detection results.

## 6. Processing Method When the Structured Light Source is Enabled

**Figure 14:**
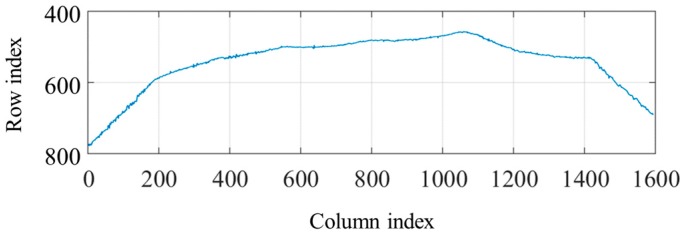
The laser stripe curve with one pixel width after thresholding and region thinning.

When the structured light source is on, the thresholding and region thinning methods are at first used to obtain the laser stripe curve with one pixel width, as shown in Figure 14. The threshold is set to 30% of the maximum grayscale values in the image. After thresholding, the region thinning algorithm is applied by finding the middle point of the longest segment in each column.

**Figure 15:**
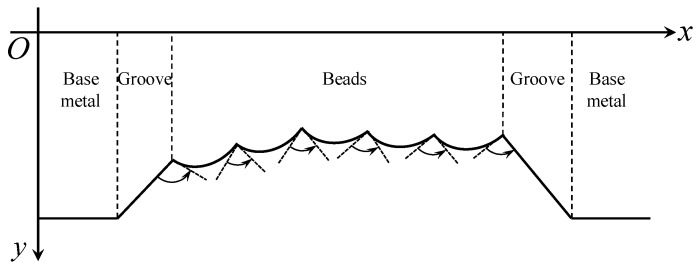
The tangent slope changes of the bead borders in the laser stripe curve.

Figure 15 shows the tangent slope changes of the bead borders in the laser stripe curve. It can be concluded that the borders correspond to the positions where the slopes increase most rapidly, i.e., the second derivative values are positive and reach the local maximums.

**Figure 16:**
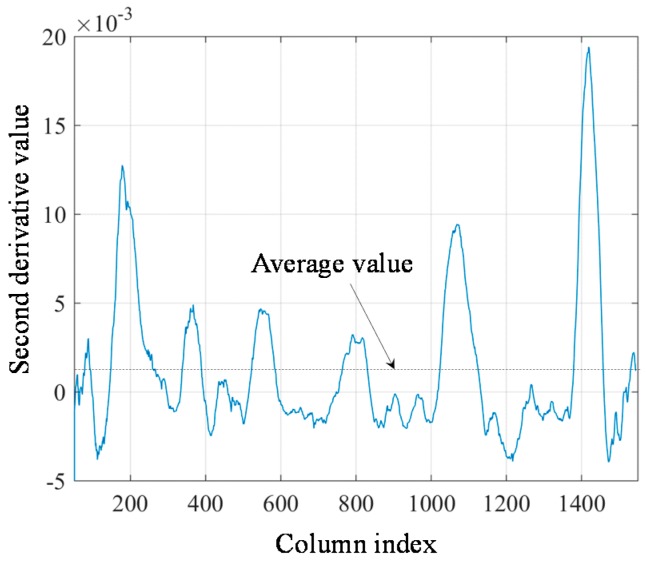
The second derivative values of the laser stripe curve calculated by Savitzky-Golay filter.

Considering that the traditional finite difference methods are sensitive to noises, a 3-order 101-point Savitzky-Golay filter is used to calculate the second derivative values of the laser stripe curve in the study. As the Savitzky-Golay filter is an FIR filter, the FFT algorithm can be used to calculate the second derivative values fast. The filter length is set to 101 because the minimum weld pass is more than 50 pixels as mentioned in Section 5. The calculation results of the second derivative values are shown in Figure 16.

**Figure 17:**
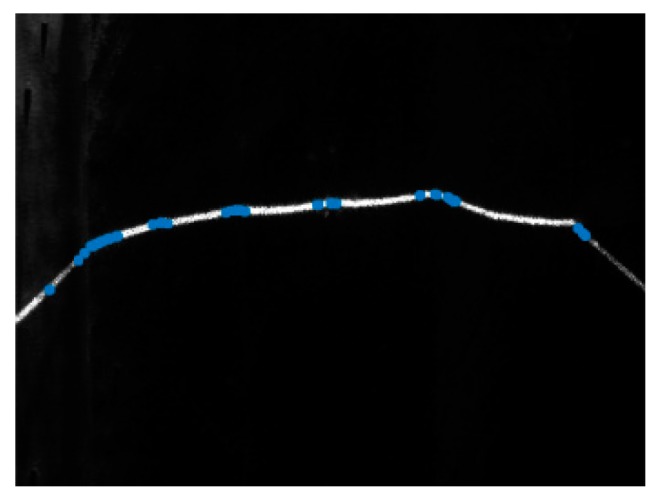
The candidate positions of the actual borders (marked in blue) based on the structured light image.

Setting the threshold *T* to the average value of the second derivative curve in Figure 16, it can be found that all of the positive peaks exist where the second derivative values are larger than *T*. There are 45 positive peaks left after thresholding and they are marked in blue in Figure 17. These remaining positive peaks are considered as the candidate positions of the actual borders.

## 7. Information Fusion Method for Directional and Structured Light Images

**Figure 18:**
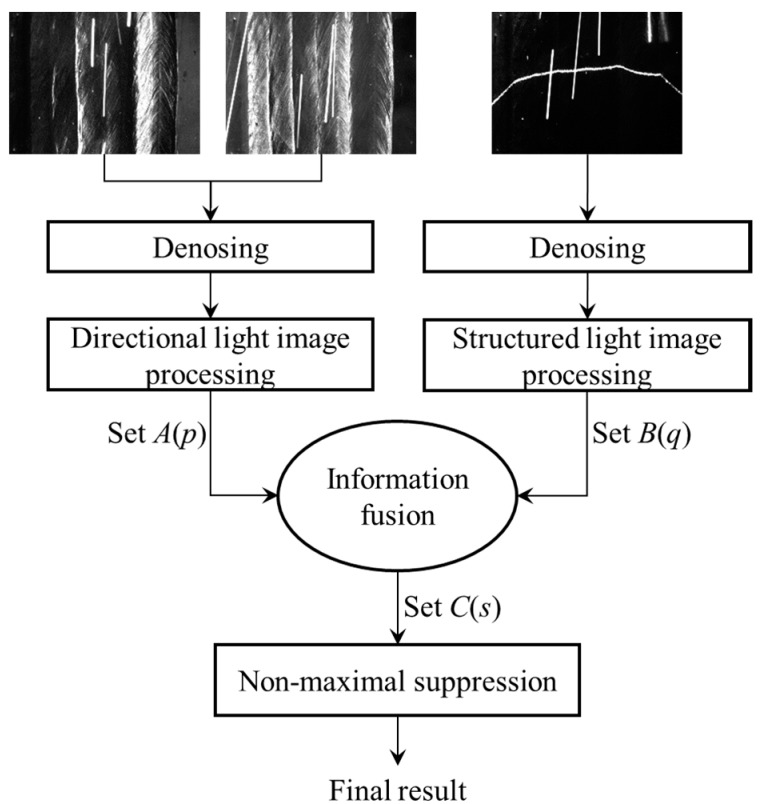
The processing algorithm fusing the images when directional light sources and structured light source are on respectively.

As demonstrated in previous sections, when the directional light source is enabled, the approximate 2D positions of the bead borders can be found, but there are still some detection errors; when the structured light source is enabled, some candidate positions of the bead borders can be obtained, but there are too many candidate points to distinguish the actual borders. A weld position detection method is proposed which fuses these two kinds of visual information. The flow of the algorithm is shown in Figure 18, and the steps of the processing algorithm are detailed as follows:

(1) Denosing. When *L*_1_ or *L*_2_ are enabled, Equation (2) is used to eliminate the arc light and spatters in the images; when *L*_3_ is enabled, Equation (1) is used instead to eliminate the arc light and spatters.
(2) Directional light image processing. First, the processing method proposed in Section 5 is used to calculate the curve of the largest gradient point numbers, as shown in Figure 13. Then, all of the valid peaks in Figure 13 are found using the thresholding and non-maximum suppression method as illustrated in Section 5. Denote the set containing all of the valid peaks by *A*(*p*). For each valid peak point *p_i_* in *A*(*p*), record its confidence interval [*p_i_* − *l_i_*, *p_i_* + *r_i_*], in which the values of the curve in Figure 13 are not less than 50% of the peak values.
(3) Structured light image processing. As mentioned in Section 6, the second derivative values of the laser stripe curve are calculated, and the candidate points are found using thresholding method. Denote the set containing all of the candidate points by *B*(*q*). Record the second derivative value *d_q_*_,*j*_ at each point *q_j_*.
(4) Information fusion. The actual positions of the borders are expected to lie in the confidence interval of the set *A*(*p*) and belong to the set *B*(*q*). For each element *p_i_* in *A*(*p*), detect whether there is any element in *B*(*q*) that is located in the confidence interval [*p_i_* − *l_i_*, *p_i_* + *r_i_*]. If these elements exist in *B*(*q*), the element *s_k_* with largest second derivative value *d_p_*_,*j*_ is most likely to be the actual position of the border; if not, just ignore *p_i_*. After these processing steps, a new candidate point set *C*(*s*) containing all *s_k_* can be obtained.
(5) Non-maximum suppression. The non-maximum suppression algorithm is applied to the set *C*(*s*), eliminating the elements close to each other. For the cases studied in this paper, the distance threshold of non-maximum suppression process is set to 50 pixels. The final detection result is recorded in the set *C*(*s*) after non-maximum suppression.

**Figure 19:**
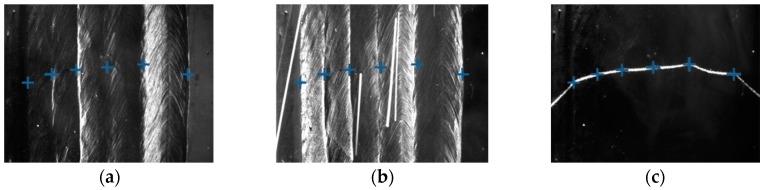
The detection results marked in different images. (**a**) Results marked in the image when the left-side DLS is on; (**b**) Results marked in the image when the right-side DLS is on; (**c**) Results marked in the image when the SLS is on.

The step (2) and (3) of the proposed algorithm can run in parallel using different threads. Figure 19 shows the final detection results using the algorithm. The actual positions of the bead borders have been accurately detected.

In addition, the 3D positions of the borders can be calculated using the structured light information. Suppose that ***γ*** is the pixel coordinate of any point on any detected border. According to the pinhole model of the camera [36], the corresponding 3D coordinate ***Γ*** to ***γ*** in the camera coordinate system can be calculated by:

(3) $$\Gamma =z_{C}\cdot S\left(\gamma \right),$$

where *z_C_* is an unknown parameter and ***S***(***γ***) can be expressed as:

(4) $$S\left(\gamma \right)=\left[\begin{array}{c} f_{1}\left(\gamma \right)\\ f_{2}\;\left(\gamma \right)\\ 1 \end{array}\right],$$

where *f*_1_ and *f*_2_ are the mapping functions from pixel coordinate to 3D coordinate, which are both determined by the intrinsic parameters of the camera. ***S***(***γ***) has no closed-form expression when the lens distortion cannot be omitted. But each ***S***(***γ***) of each pixel in the image can be calculated beforehand and a lookup table for real-time calculation can be created.

Suppose the light plane equation of the structured light in the camera coordinate system is:

(5) $$n^{T}X=c$$

where ***X*** is any point in the light plane, ***n*** is the unit normal vector of the light plane, and *c* is the directed distance between the light plane and the origin of the camera coordinate system. ***n*** and *c* can be both calibrated beforehand.

The 3D coordinate ***Γ*** meets (5) since ***Γ*** is located in the light plane of the structured light. Combining (3) and (5), the 3D coordinate ***Γ*** can be calculate by:

(6) $$\Gamma =\;\frac{c}{n^{T}S\left(\gamma \right)}S\left(\gamma \right)$$

using (6), the 3D positions of the bead borders can be obtained.

## 8. Experiments and Discussions

**Figure 20:**
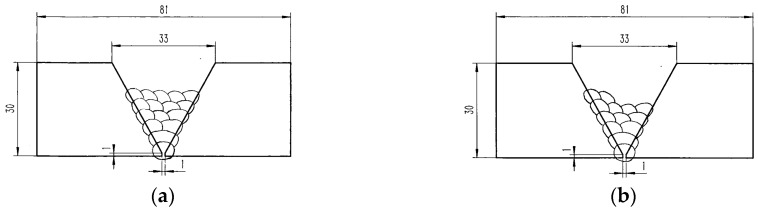
Workpiece samples. (**a**) Sample A with five visible passes in the same layers; (**b**) Sample B with five visible passes in different layers.

Two kinds of workpiece samples used in this paper are shown in Figure 20, namely sample A and sample B. Samples A and B have both five visible passes on their top surfaces. The five visible passes in sample A are all located in the same layer. In sample B, the five visible passes are not all located in the same layer: two passes are located in the 6th layer while the other three are located in the 5th layer. The shielding gas during welding is argon or CO_2_. The materials of the samples are low carbon steel.

**Figure 21:**
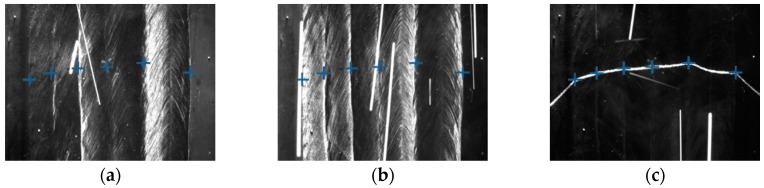
The experiment results when the workpiece is sample A and the shielding gas is argon. (**a**) Results marked in the image when the left-side DLS enabled; (**b**) Results marked in the image when the right-side DLS enabled; (**c**) Results marked in the image when the SLS enabled.

**Figure 22:**
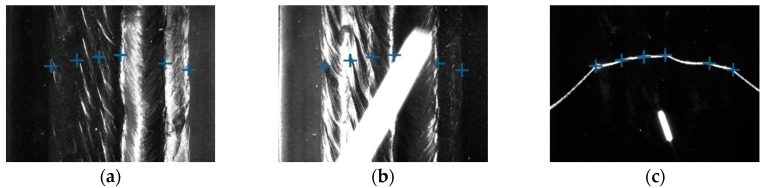
The experiment results when the workpiece is sample B and the shielding gas is argon. (**a**) Results marked in the image when the left-side DLS enabled; (**b**) Results marked in the image when the right-side DLS enabled; (**c**) Results marked in the image when the SLS enabled.

**Figure 23:**
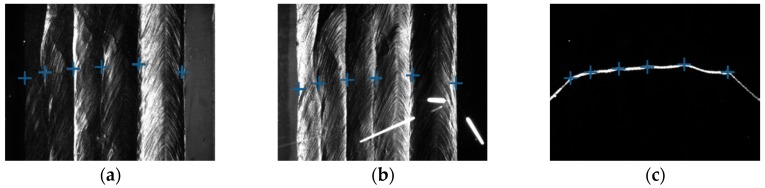
The experiment results when the workpiece is sample A and the shielding gas is CO_2_. (**a**) Results marked in the image when the left-side DLS enabled; (**b**) Results marked in the image when the right-side DLS enabled; (**c**) Results marked in the image when the SLS enabled.

**Figure 24:**
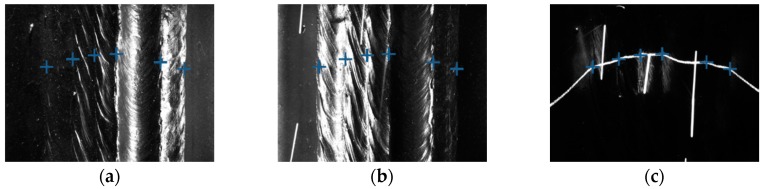
The experiment results when the workpiece is sample B and the shielding gas is CO_2_. (**a**) Results marked in the image when the left-side DLS enabled; (**b**) Results marked in the image when the right-side DLS enabled; (**c**) Results marked in the image when the SLS enabled.

**Figure 25:**
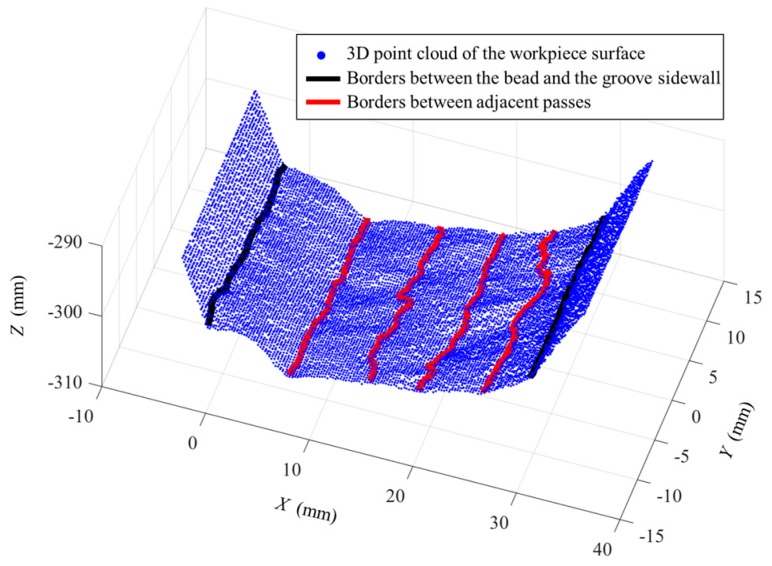
3D reconstruction results of weld pass positions when the workpiece is sample A and the shielding gas is argon.

**Figure 26:**
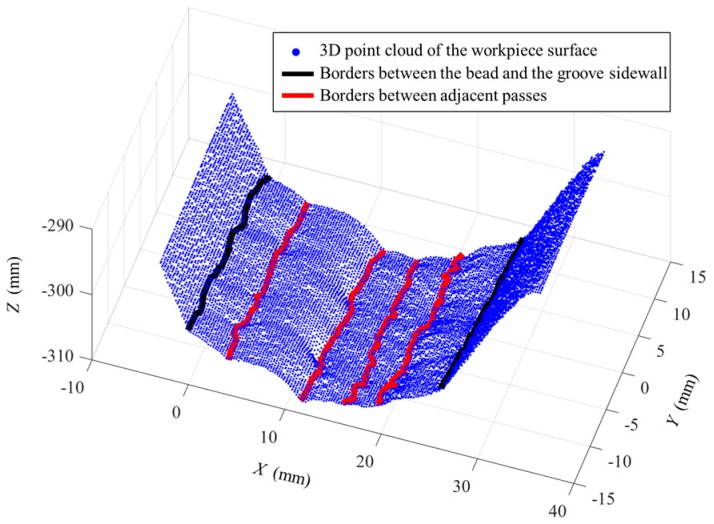
3D reconstruction results of weld pass positions when the workpiece is sample B and the shielding gas is argon.

**Figure 27:**
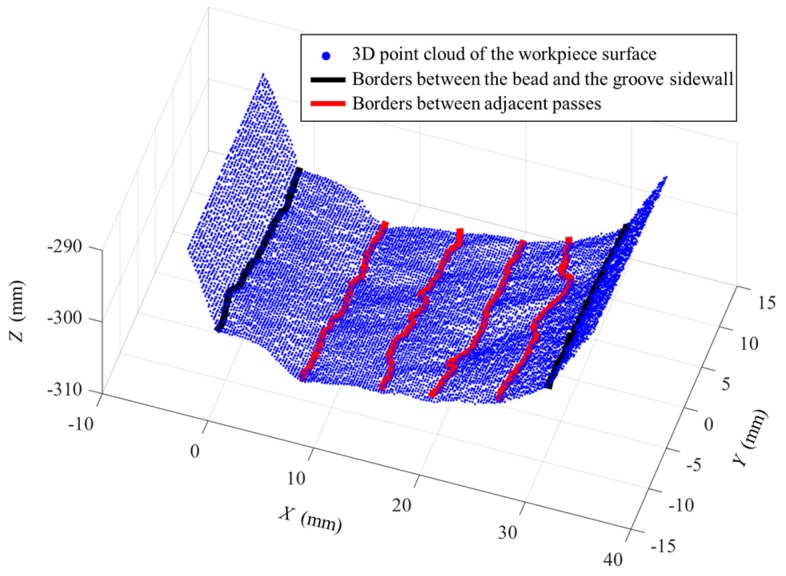
3D reconstruction results of weld pass positions when the workpiece is sample A and the shielding gas is CO_2_.

**Figure 28:**
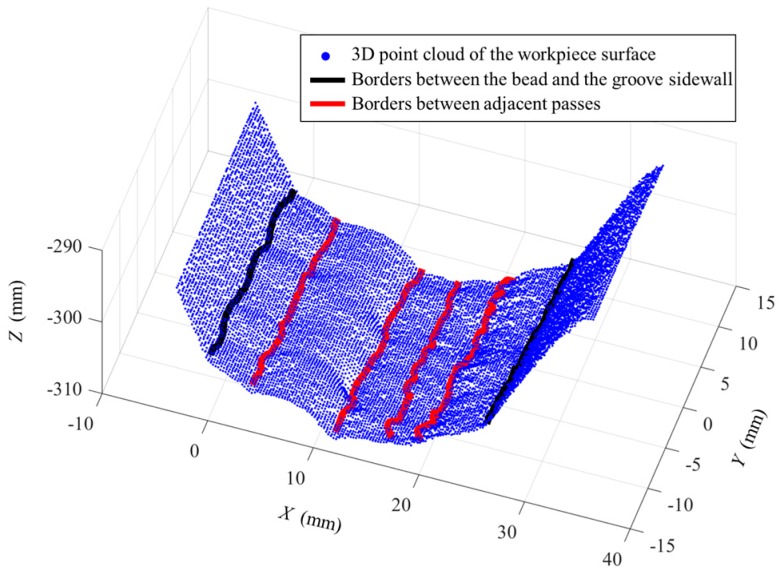
3D reconstruction results of weld pass positions when the workpiece is sample B and the shielding gas is CO_2_.

Some of the images captured during welding process are shown in Figure 21 through Figure 24. The shielding gas is argon for the welds shown in Figure 21 and Figure 22, while the shielding gas is CO_2_ for the welds shown in Figure 23 and Figure 24. Sample A is used in the experiments shown in Figure 21 and Figure 23, while the sample B is in the experiments shown in Figure 22 and Figure 24. The welding speed is set to 240 mm/min. The welding current is set to 220 A when the shielding gas is argon, and set to 120 A when the shielding gas is CO_2_. The frame rate and image size of the camera are set to 30 fps and 1200 × 1600. The field of view is about 25 mm × 33 mm, which indicates that the image resolution is about 0.021 mm/pixel. Figure 25, Figure 26, Figure 27 and Figure 28 show the 3D reconstruction results of the MLMPW path. Figure 25, Figure 26, Figure 27 and Figure 28 correspond to the experiments in Figure 21, Figure 22, Figure 23 and Figure 24, respectively.

**Figure 29:**
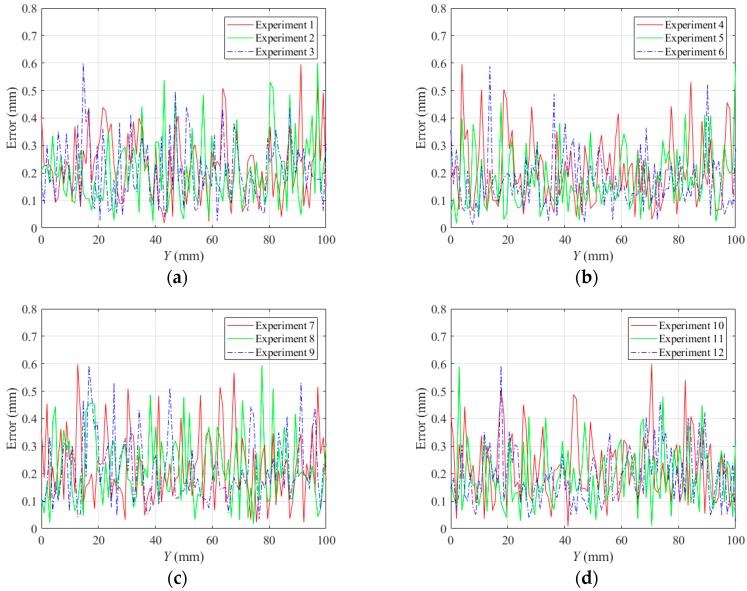

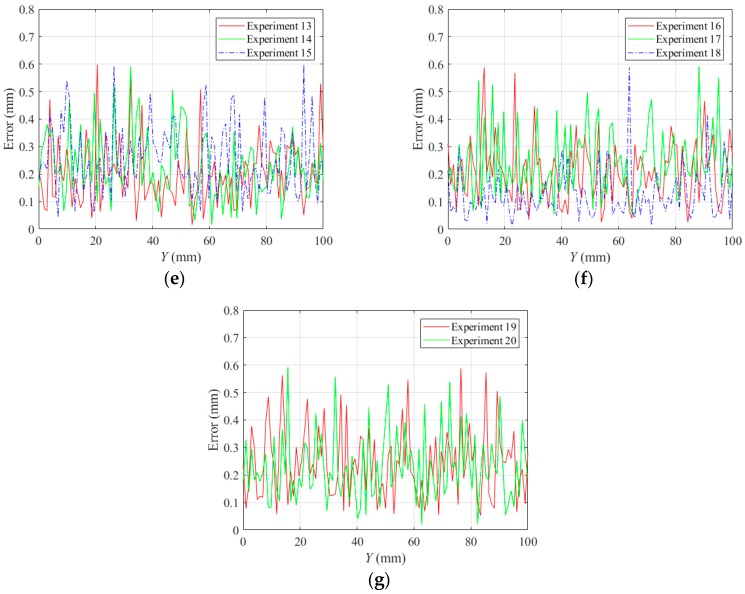
Detection error curves of the twenty experiments. (**a**) Experiments 1 to 3; (**b**) Experiments 4 to 6; (**c**) Experiments 7 to 9; (**d**) Experiments 10 to 12; (**e**) Experiments 13 to 15; (**f**) Experiments 16 to 18; (**g**) Experiments 19 to 20.

**Figure 30:**
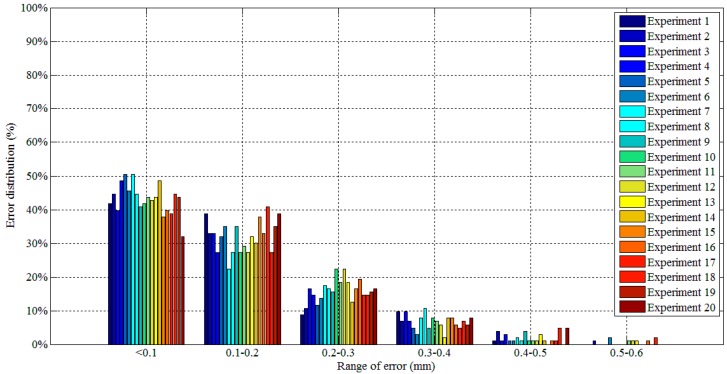
Detection error distribution of the twenty experiments.

The lines in black indicate the detection results of the borders between the bead and the groove sidewall, and the lines in red indicate the detection results of the borders between adjacent passes. Although there are five visible passes in both sample A and sample B, the passes are in different layers. The 3D reconstruction results show that these passes in different layers can be successfully distinguished. Another twenty experiments are carried out to verify the applicability of the proposed method using the same types of samples. The minimum protrusion of the bead surface in the samples is not more than 0.3 mm. We define the detection errors to be the 3D distances between the detected weld border points and the actual weld border points (the 2D positions of the actual weld border points are manually marked in the images, so that their 3D positions can be calculated by the triangulation methods using the structured light images). The detection errors of these twenty experiments are shown in Figure 29, in which *Y* means the length along the welding direction. The detection error distributions of these experiments are shown in Figure 30. Figure 29 and Figure 30 show that the maximum detection error does not exceed 0.6 mm in these experiments.

The image processing time cost of each step in our proposed algorithm is measured by the C++ function in the software program. The measured results are as follows: (1) not more than 14 ms when eliminating the arc light and spatters in three kinds of images; (2) not more than 8 ms when processing the directional light images after denosing, and not more than 5 ms when processing the structured light image after denosing; (3) not more than 3 ms when performing information fusion and the final non-maximal suppression step in Figure 18. As a result, the total time cost does not exceed 30 ms each time. All of these experimental results indicate that the proposed method can be applied in accurate and real-time 3D weld position detection during MLMPW processes.

Although the experiments in this paper is carried out using the MIG/MAG welding method, the proposed method may be also used in GTAW process, since there are less spatters and the illumination of the arc is more stable than MIG/MAG. Future research works will focus on designing a more practical and compact sensor, and performing more experiments (considering different welding method, parameters, materials, etc.) to evaluate our proposed method further.

## 9. Conclusions

This paper proposes a weld position recognition method based on directional light and structured light information fusion during multi-layer/multi-pass welding. The proposed synchronous acquisition method can make the two directional light sources and one structured light source switched on alternately; in the meanwhile, the camera can be synchronized to capture images when each light is enabled. Different images can be obtained in different lighting conditions using this stroboscopic method. In order to eliminate the interferences from the arc light and spatters, an information fusion process at pixel level are performed for the adjacent images. Afterwards, the approximate positions of the bead borders can be calculated in the directional lighting condition, while some candidate points of the bead borders can be obtained using the second derivative values of the structured light curve. Finally, a 3D weld position detection method is proposed which fuses the information from directional and structured lighting conditions, and can detect the bead borders accurately. Experiments are carried out to verify the applicability of the proposed method. The results show that the time cost of the processing algorithm is not more than 30 ms and the detection error does not exceed 0.6 mm. These research works indicate that the proposed method is applicable in online path planning and real-time seam tracking during multi-layer/multi-pass welding process as well as electron beam freeform fabrication process.

## Figures and Tables

**Table 1:** Image processing method researches using the structured light detection method.

Image Processing Methods	Detailed Research Works
Image pre-processing methods for denoising	average filtering [14], median filtering [15,16], Gaussian filtering [12], morphological filtering [17], arc light and spatters elimination using multiple frames [18,19], filtering using color space conversion to utilize the specific color information in RGB image [14], etc.
Laser stripe pattern extraction methods	global or local thresholding method [14,16], projection method [18,20], statistical method [21], laser line detection method using Hough transform [17,22], finding the pixel or sub-pixel coordinates of the laser stripe by searching the maximum grayscale position in each row/column of the image [23,24], etc.
Welding joint feature extraction and profiling methods	turning angle computation method [9,25], derivative method [23,26], rule-based method [27], corner point detection method [15], custom pixel-to-pixel operation method [11,16], etc.
